# Impact of Eye and Breast Shielding on Organ Doses During Cervical Spine Radiography: Design and Validation of MIRD Computational Phantom

**DOI:** 10.3389/fpubh.2021.751577

**Published:** 2021-10-22

**Authors:** Wiam Elshami, Huseyin Ozan Tekin, Shams A. M. Issa, Mohamed M. Abuzaid, Hesham M. H. Zakaly, Bashar Issa, Antoaneta Ene

**Affiliations:** ^1^Department of Medical Diagnostic Imaging, College of Health Sciences, University of Sharjah, Sharjah, United Arab Emirates; ^2^Physics Department, Faculty of Science, University of Tabuk, Tabuk, Saudi Arabia; ^3^Physics Department, Faculty of Science, Al-Azhar University, Cairo, Egypt; ^4^Department of Experimental Physics, Institute of Physics and Technology, Ural Federal University, Yekaterinburg, Russia; ^5^Department of Chemistry, Physics and Environment, Faculty of Sciences and Environment, INPOLDE Research Center, Dunarea de Jos University of Galati, Galati, Romania

**Keywords:** dose reduction, radiation protection, shielding, monte carlo method, MCNPX

## Abstract

**Purpose:** The study aimed to design and validate computational phantoms (MIRD) using the MCNPX code to assess the impact of shielding on organ doses.

**Method:** To validate the optimized phantom, the obtained results were compared with experimental results. The validation of the optimized MIRD phantom was provided by using the results of a previous anthropomorphic phantom study. MIRD phantom was designed by considering the parameters used in the anthropomorphic phantom study. A test simulation was performed to compare the dose reduction percentages (%) between the experimental anthropomorphic phantom study and the MCNPX-MIRD phantom. The simulation was performed twice, with and without shielding materials, using the same number and locations of the detector.

**Results:** The absorbed dose amounts were directly extracted from the required organ and tissue cell parts of output files. Dose reduction percentages between the simulation with shielding and simulation without shielding were compared. The highest dose reduction was noted in the thymus (95%) and breasts (88%). The obtained dose reduction percentages between the anthropomorphic phantom study and the MCNPX-MIRD phantom were highly consistent and correlated values with experimental anthropomorphic data. Both methods showed Relative Difference (%) ranges between 0.88 and 2.22. Moreover, the MCNPX-MIRD optimized phantom provides detailed dose analysis for target and non-target organs and can be used to assess the efficiency of shielding in radiological examination.

**Conclusion:** Shielding breasts and eyes during cervical radiography reduced the radiation dose to many organs. The decision to not shield patients should be based on research evidence as this approach does not apply to all cases.

## Introduction

Diagnostic radiology is associated with a high collective radiation dose (1), and a patient's exposure to diagnostic ionizing radiation is associated with an increased risk of cancer (2). The International Commission on Radiological Protection (ICRP) recommendation is to optimize and limit exposure to ionizing radiation when possible. Efforts should be made to ensure patient safety and reduce radiation exposure to as low as reasonably achievable (ALARA) while ensuring adequate image quality (3, 4).

Lead (Pb) shields are commonly used to protect patients during radiology procedures (5, 6). The importance of the shielding materials for medical and industrial applications was highlighted by different investigations (7–10). In particular, a remarkable reduction in absorbed radiation dose for testes, ovaries, eyes, and breasts was observed via experimental studies that utilized shielding during radiology procedures. Experimental studies used an anthropomorphic phantom to assess radiation dose for target and non-target organs. Target organs are situated in the primary field of radiation. Even though non-target organs are away from the field of radiation, they were also affected by the scatter radiation (9–13).

Despite these findings, recent studies suggest that shielding patients has no benefits and should be discontinued as the radiation-absorbed dose from a single examination is mostly negligible (14, 15). The major concern is the cumulative risk from multiple radiological examinations, particularly for children and young patients (16), and the decision to not shield patients should be based on research evidence as one size does not fit all (9). Calculation of absorbed radiation doses has an essential place in radiation medical applications to support patient safety and reduce the risk of radiation injuries. Although it is possible to calculate non-target doses in some cases, it is physically very difficult or impossible in many cases. Therefore, the major limitation of using the anthropomorphic phantom in dose measurement studies was to observe the variety of absorbed radiation dose in organs and tissue at micro-scale dosimetry.

In recent years scientists use simulation methods to overcome such situations. One of the most powerful methods used in radiation calculations is Monte Carlo (MC) simulations, which are commonly used in medical radiation physics (17–19). This is a theoretical model in which physical quantities are determined by simulating the transport of X-ray photons. A wide variety of MC codes are available in the literature for various forms of medical radiation application. However, some of them, such as Geant4 (20), PENELOPE (21), MCNPX (22), FLUKA (23), and EGSnrc (24), are even more prominent due to their ease of use, rich libraries, and consistent results. Initial efforts to shape an individual phantom to measure radiation absorbed doses were performed by Cristy et al. (25). Then, different types of human computational phantoms, including the MIRD (Medical Internal Dose) phantom (26, 27), were used for medical radiation measurements by many researchers (28–32).

The literature clearly outlined that MC codes and computational phantoms can model high-precision radiation transport within matter and be implemented to estimate the absorbed radiation dose in medical applications. This has encouraged many authors to perform an extensive organ dose assessment using MCNPX (Monte Carlo N-Particle Transport Code) and MIRD computational phantom (25–33). To the best of our knowledge, no study explored the efficiency of shielding in dose reduction using the computational phantom and MCNPX codes. Optimization of the computational phantoms using the MCNPX code could provide opportunities to simulate the required imaging technique, patient position, radiation sources and direction, and shielding materials. Therefore, it is expected to provide a remarkable contribution to enrich the radiology and medical physics literature about the role of shielding in protecting patients from unnecessary radiation. The current study aimed to design and validate computational phantoms (MIRD) using the MCNPX code to assess the impact of shielding on organ doses during radiology examinations.

## Materials and Methods

The study aimed to determine the impact of shielding on organ and tissue dose during radiology examinations using MCNPX code and MIRD Phantom. To address this aim, the study designed a computational phantom (MIRD) using the MCNPX code and then validated it using an anthropomorphic phantom experiment.

### Monte Carlo Simulations and MCNPX Code

MCNPX (***version 2.7.0***) (22) computer simulations were conducted on a voxelized MIRD phantom mentioned in the International Commission on Radiological Protection (ICRP) Publication 110, which describes the features of an adult reference female in ICRP Publication 74 (34). ICRP's phantoms are mathematical representations of the anatomy of the human body, which are used in dosimetry measurements. The advances that have been made to these phantoms, especially in the last few years, represent substantial advances in scientific methods and analytical capacity, along with changing perceptions that the best evidence can be used and shared publicly. The sophisticated models presented in this publication will allow the Commission to be prepared for the potential calculations needed.

**Figure 1:**
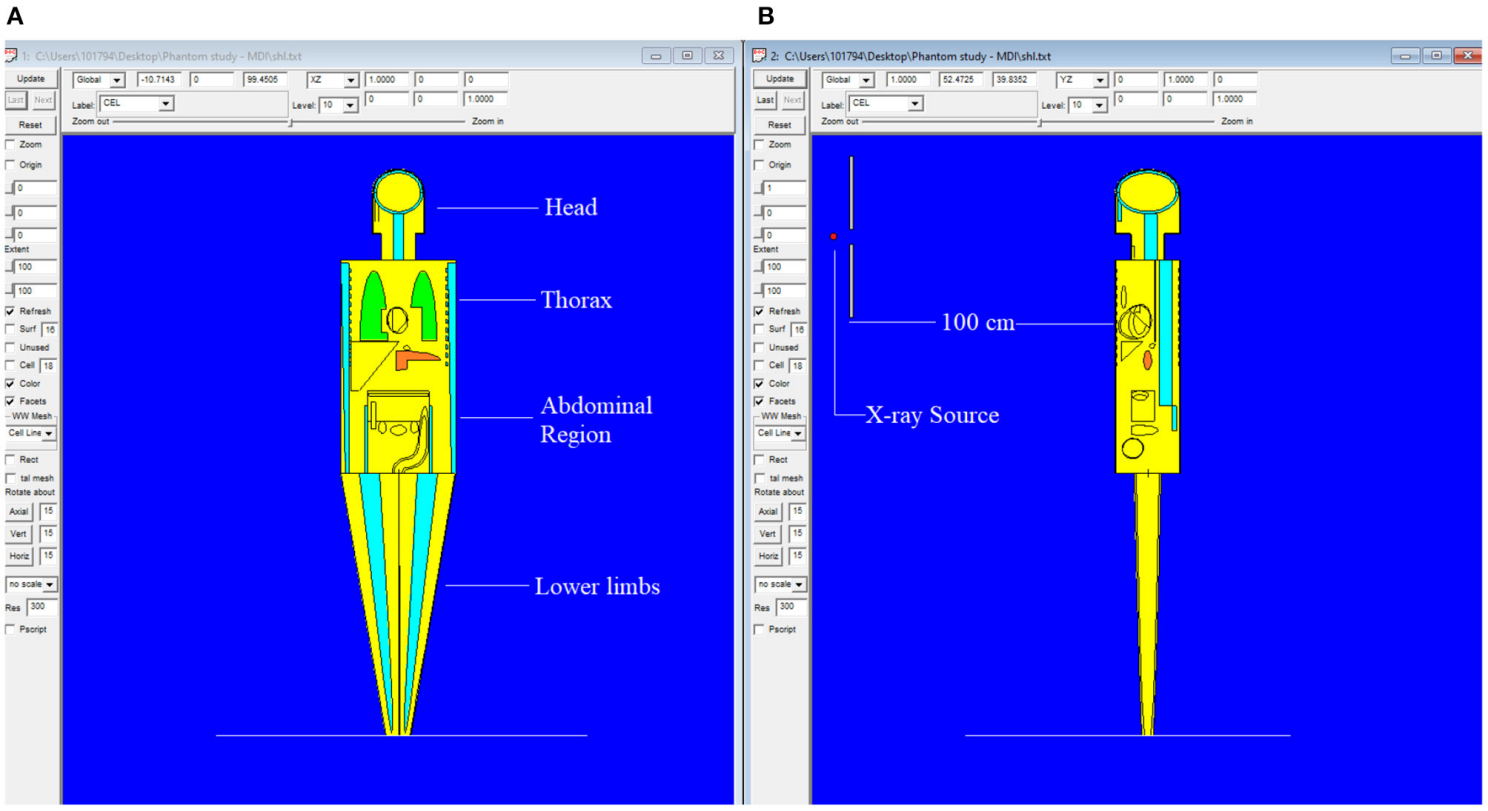
MIRD phantom utilized in this research using MCNPX general purpose Monte Carlo code (***version 2.7.0***) **(A)** Antero-posterior view of MIRD phantom **(B)** Lateral view of MIRD phantom.

**Figure 2:**
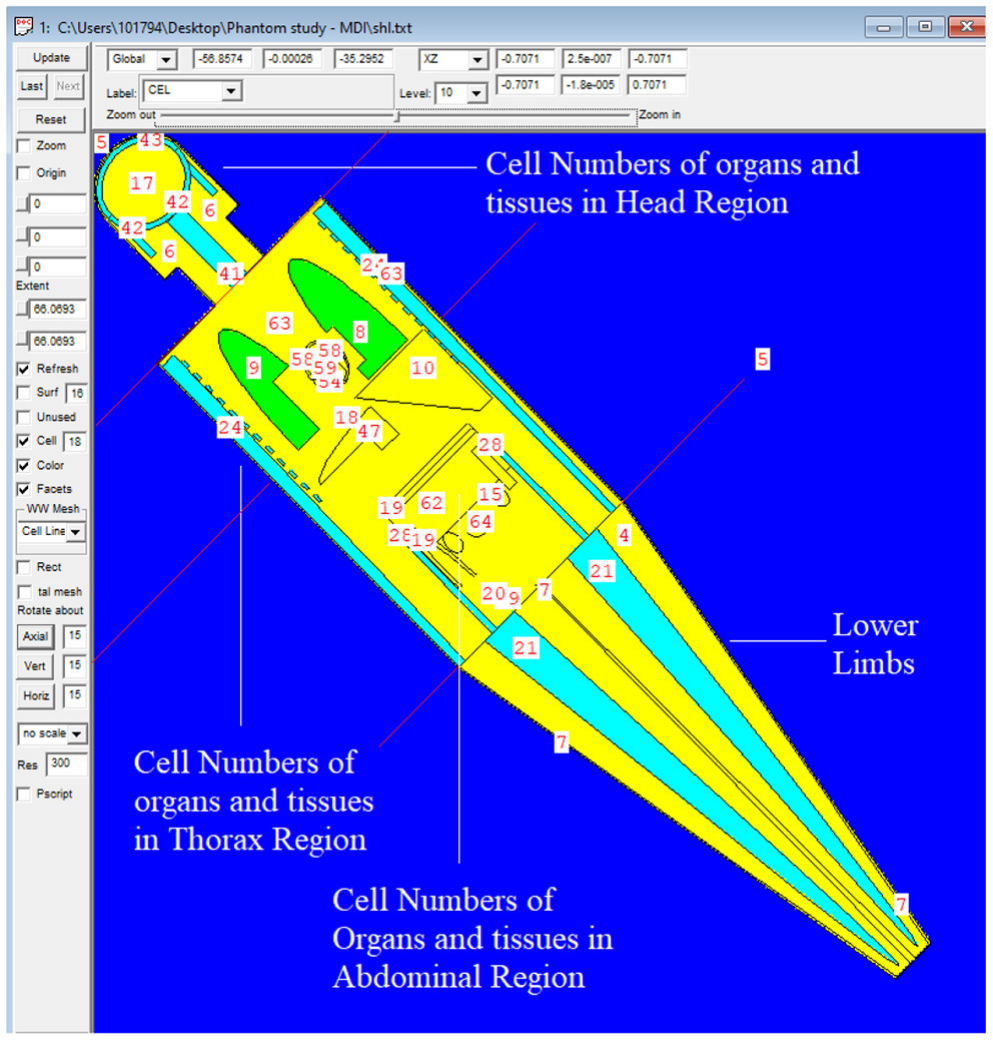
General appearance of the modified MIRD phantom and CELL numbers of the organs and tissues.

**Figure 3:**
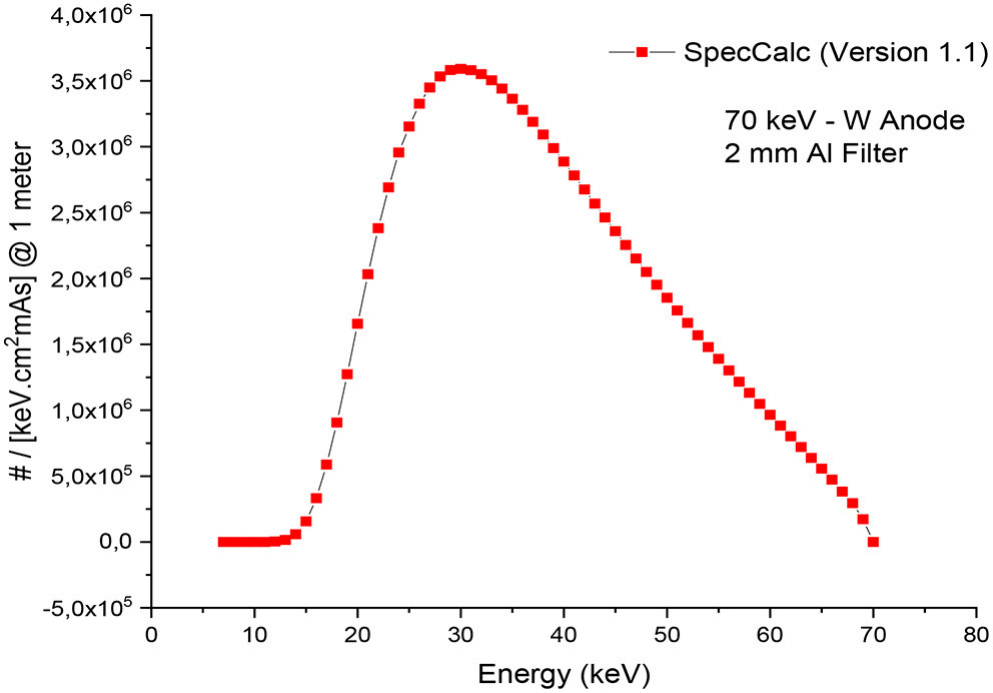
X-ray spectrum obtained from SpecCalc (*version 1.1*) for 70 kVp tube voltage and 2 mm Al Filter.

The MIRD, known as a computational phantom, has been defined to represent the structure of a human body with essential properties such as organs, tissues, elemental mass fractions, and densities. The initial heterogeneous MIRD has been published by OAK Ridge National Laboratory (ORNL). During the last few decades, the MIRD phantom has been updated, and many organizations such as NRC (Nuclear Regulatory Commission) used this phantom as a reference for different types of investigations. In the current study, ORNL-MIRD (1996) has been used for extensive organ dose evaluations during radiology examinations. The MIRD phantom has been optimized by considering radiology examination conditions (i.e., patient-source distance, radiation source energy, definitions of body parts, the geometry of shields). The structure of the MIRD phantom with its cell and surface details can be seen in Figure 1A. Figure 1B shows the position of the X-ray source from the lateral view of the MIRD phantom. The phantom represents an adult female body that can be used for many radiology examinations in erect and supine positions. The MIRD surfaces are reconstructed by MCNPX surface and cell definitions. The general appearance of the modified MIRD phantom and CELL numbers of the organs and tissues are seen in Figure 2. Initially, we used the F6 Tally Mesh length estimator to measure the energy deposition, and when they were the same, we use the ^*^f8 tally, which absorbed score doses in terms of MeV in a cell-based on secondary electron dose depositions. No photon and electron energy cutoffs were used in our simulations. As a first step, the MCNPX code was run for 10^8^ histories or NPS (number of particle history) without any shield protection on the MIRD phantom. The uncertainty of MC estimations was <1% in all simulations. The source definition has been done in the ***sdef*** (source definition) part of the INPUT file. Accordingly, ***si*** (source probability) and ***sp*** (source bias) variables were defined by considering the beam distribution of the X-ray from the source. The source consisted of a point source with a directional bias toward the phantom according to realistic geometry used for radiography. The beam size was 18 × 24 cm on the patient surface. The angle of direction bias was 5.14 degrees and was defined in the SDEF card. The photon beam energy spectra of a radiography tube were derived from SpekCalc (version.1) (35–37) considering a 2 mm intrinsic Aluminum filter according, to the tube manufacturer's instructions. The literature review showed that similar investigations were performed using SpeCalc to provide suitable X-ray spectra for the source definition of MCNP code (38–40). The photon beam spectra for 70 kVp are depicted in Figure 3. The obtained normalized spectra were utilized to provide the energy-histogram to define the source definition card (SDEF). It was selected as a point isotropic source with a histogram of energy (Point Isotropic Source with a Histogram of Energies). However, it was collimated onto the MIRD phantom, where X-ray angle was calculated to fit exactly the diameter of the experimental study.

### Validation of MCNPX Code Through Experimental Data

**Figure 4:**
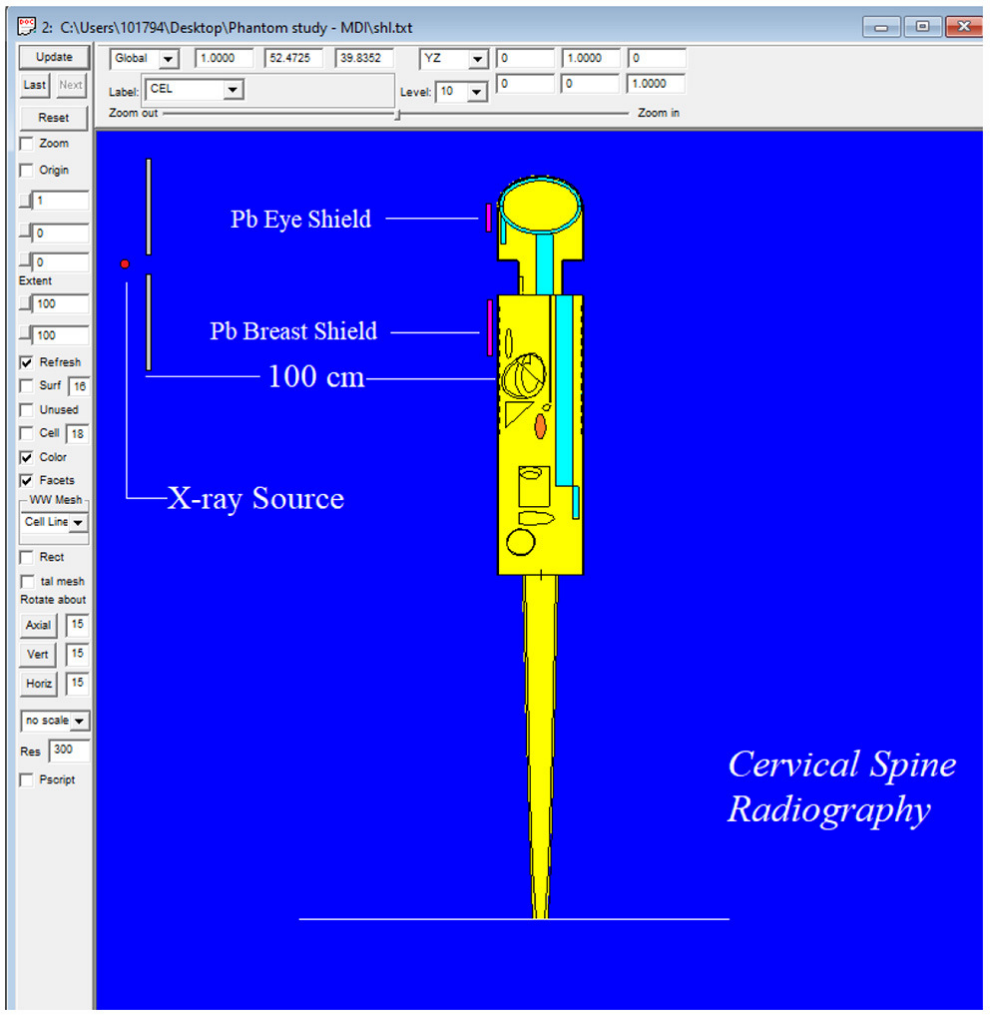
The modified MIRD phantom, shielding materials, and their locations.

To validate the outcomes of the MCNPX code and the optimized MIRD phantom, MCNPX-MIRD phantom, previous experimental results from an anthropomorphic phantom study were used. The study assessed the radiation dose absorbed by eyes and breasts during Antero-Posterior (AP) cervical radiography using anthropomorphic phantom (9). In the current study, the technique and exposure parameters used in the anthropomorphic phantom experiment (AP projection, fine focus, kVp = 70, and focus detector distance = 100 cm) were replicated in the MCNPX-MIRD phantom. Similarly, the clinical conditions such as the patient in an erect position, x-ray source, and shielding type, shielding thicknesses, and shielding locations were determined similar to the anthropomorphic phantom study. The definition of shielding materials was implemented in the material definition part of the INPUT file. Moreover, the geometrical properties of shielding materials with their material thicknesses have been defined in the surface cards. Finally, two different lead (Pb) shield materials were defined in the MCNPX-MIRD phantom as the same as in the anthropomorphic study. The MCNPX-MIRD phantom with the added shielding materials. Their locations can be seen in Figure 4.

A test simulation was performed to compare the dose reduction percentages (%) between the experimental anthropomorphic phantom study and the MCNPX-MIRD phantom. The simulation was performed twice, with and without shielding materials, using the same number and locations of the detector. Accordingly, the energy deposition amounts of each cell (Figure 2) were obtained using the F6 tally mesh of MCNPX. The scored absorbed dose by MCNPX was the averaged dose over cell volume. F6 Tally calculates the dose in terms of MeV/g per initial photon. This type of tally can provide the average energy deposition amount in a certain cell. Finally, the required transformations from recorded data to experimental data were done using verified methods for MCNPX 2.7.0 and MCNP-4C codes (41–43). The results were recorded in the output file, and accordingly, the absorbed dose amount in each pre-defined cell was exported. Table 1 shows the names of organs and their cell number codes.

**Table 1:** Energy depositions (MeV/g per initial photon) of some organs and tissues obtained from MCNPX output (F6 Tally Mesh).

**Organ/**	**Cell**	**Without shielding**	**With shielding**
**tissue**	**number**	**material**	**material**
Skin	7	2.8871e-16	2.5859e-16
Lung	8	1.7134e-07	1.0228e-07
Liver	10	1.8291e-07	1.7651e-07
Stomach	11	8.0897e-10	7.9340e-10
Urinary bladder	13	4.4353e-10	4.4520e-10
Ovaries	15	8.9999e-08	9.0950e-08
Brain	17	1.2332e-07	8.9033e-08
Esophagus	18	1.0537e-07	8.0153e-08
Colon	19	3.4818e-10	3.4912e-05
Leg bones	21	3.3325e-08	3.3361e-08
Arm bones	24	1.1667e-07	9.4818e-08
Clavicles	25	2.6587e-07	2.6380e-07
Scapulae	26	4.9649e-08	3.4071e-08
Pelvis	28	4.6403e-08	4.6487e-08
Rib cage	29	1.7805e-07	1.2980e-07
Spine	41	5.2727e-08	4.7404e-08
Skull-cranium	42	6.1291e-08	2.6767e-08
Facial skeleton	43	3.1124e-07	2.5174e-07
Thyroid	44	3.5803e-07	3.5542e-07
Kidneys	45	1.2468e-10	1.1880e-10
Pancreas	47	3.5941e-10	3.3205e-10
Spleen	48	2.1856e-10	2.0249e-10
Thymus	49	9.8690e-10	3.4890e-10
Adrenals	50	1.4914e-10	1.4369e-10
Gall bladder	52	4.3957e-10	4.3677e-10
Heart-left ventricle	54	5.9281e-10	3.3882e-10
Heart-left ventricle-contents	55	5.9281e-10	3.3882e-10
Heart-right ventricle	56	5.9281e-10	3.3882e-10
Heart-right ventricle-contents	57	5.9281e-10	3.3882e-10
Heart-left atrium	58	5.9281e-10	3.3882e-10
Heart- left atrium-contents	59	5.9281e-10	3.3882e-10
Heart-right atrium	60	5.9281e-10	3.3882e-10
Heart- right atrium-contents	61	5.9281e-10	3.3882e-10
Small intestine	62	3.2101e-10	3.2094e-10
Uterus	64	2.6720e-10	2.6742e-10
Breasts	65	1.0986e-09	4.2349e-10

### Organ Dose Assessment

The radiation doses absorbed by eyes and breasts during AP cervical examination were recorded in two settings (with shielding and without shielding). Finally, a comparison of obtained dose reductions between the anthropomorphic phantom study and the optimized MIRD was performed.

## Results

**Figure 5:**
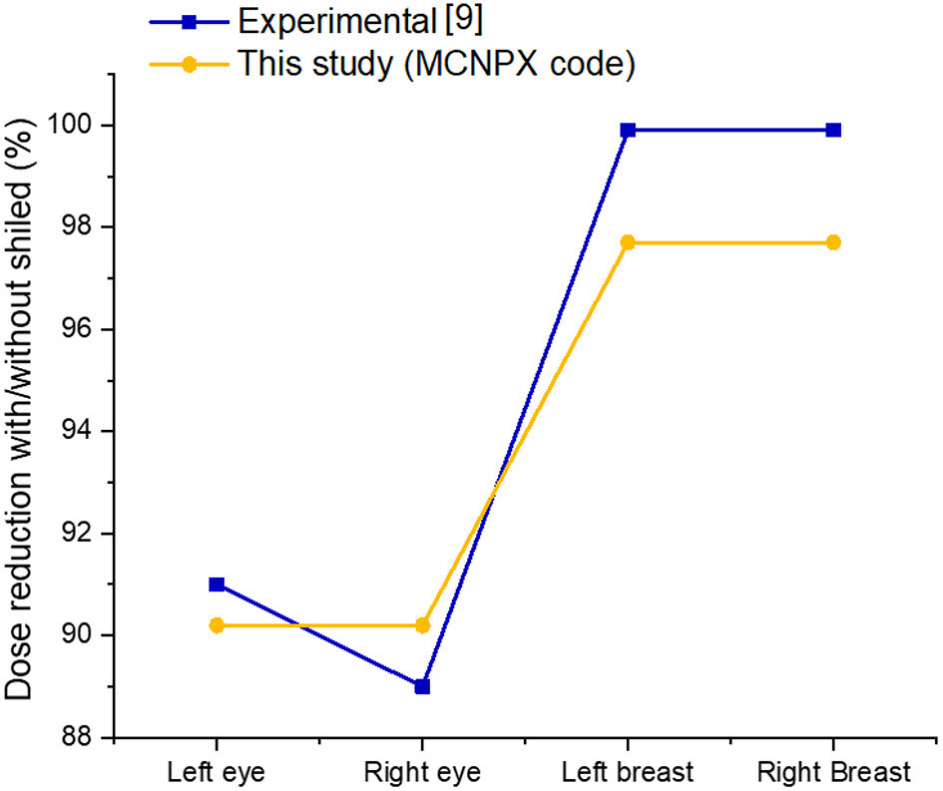
Comparison of dose reductions achieved in experimental and Monte Carlo studies.

In this study, a computational phantom (MIRD) was optimized using the MCNPX code to assess the impact of shielding on organ doses during radiology examination. A previous anthropomorphic phantom study was used to validate the results of the current study (9). The present study revealed a smooth difference in the variation of organ dose reduction percentages. As shown in Figure 5; Table 2, the obtained dose reduction percentages between the anthropomorphic phantom study and the MCNPX-MIRD phantom were reported for the left eye, right eye, left breast, and right breast. Both methods showed Relative Difference (%) ranges between 0.88 and 2.22. The outcomes obtained from the MCNPX-MIRD phantom can be considered highly consistent and the values correlated with experimental anthropomorphic data.

**Table 2:** Comparison of obtained dose reductions between experimental and Monte Carlo study.

**Organ type**	**Obtained dose reduction in Experimental study [32] (%)**	**Obtained dose reduction in this study (MCNPX code) (%)**	**Relative difference (%)**
Left eye	91	90.2	0.88
Right eye	89	90.2	1.33
Left breast	99.9	97.7	2.22
Right Breast	99.9	97.7	2.22

* Relative difference$=\frac{Experimental\;\%-MCNPX\;\%}{\left(Experimental\;\%+MCNPX\;\%\right)/2}\;\times \;\text{100}\%$.

**Figure 6:**
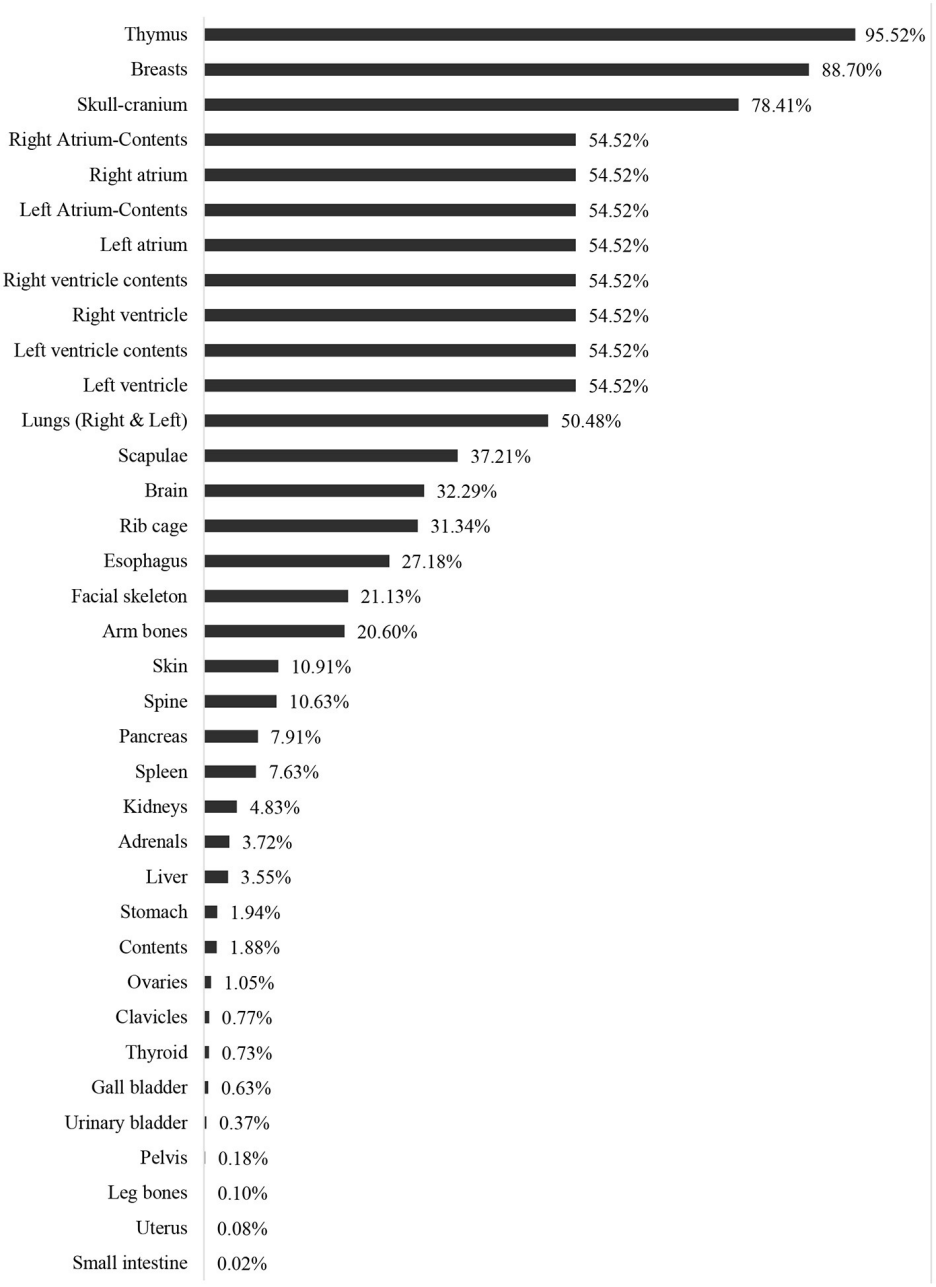
The reduction percentages (%) of the absorbed radiation doses in organs and tissues.

Shielding utilization would significantly decrease the absorbed radiation dose amount in the breasts. Moreover, the use of the MCNPX-MIRD phantom provided detailed information about dose reduction due to the shielding used at the level of the eyes and breasts (Figure 6). It is worth mentioning that it is very difficult to observe such detailed dose measurement for non-target organs using the anthropomorphic phantom.

Besides the validation, the current study revealed that the highest dose reeducation was observed in the thymus (95.52%) and breasts (88.70%). The majority of the thoracic region showed organ dose reduction such as Heart-Left Ventricle, Heart-Left Ventricle-Contents, Heart-Right Ventricle, Heart-Right Ventricle-Contents Heart-Left Atrium, Heart- Left Atrium-Contents, Heart-Right Atrium Heart- Right Atrium-Contents (Figure 5). Overall, a 54.52% dose reduction was reported with the use of shielding compared to without shielding using the MCNPX-MIRD phantom during AP cervical.

## Discussions

The present study designed and validated an MCNPX-MIRD optimized phantom. The result of the current study was compared with a previous study (9) and found that the modified phantom provides highly consistent and correlated radiation dose values with experimental anthropomorphic data. Moreover, it provides a detailed analysis of the role of shielding application in reducing organ dose during radiology examination. In contrast to a previous study investigating the effect of shielding in reducing radiation dose to gonads, breast, and eyes using dosimeters (9), the MCNPX-MIRD phantom makes it possible to assess the radiation dose to other organs and tissues. Our results demonstrate that the application of shielding reduced radiation dose to other organs that are not located in the field of primary radiation. The utilization of shielding benefits the patient by reducing the absorbed dose, and the result of the current study demonstrated a reduction of more than 95.52 and 88.70% in the thymus and breasts, respectively. Exposure of the thymus to low doses of ionizing radiation may lead to changes and disrupt the immune system's function and mechanism (44). Moreover, radiation exposure to the head and neck can result in thyroid dysfunction and may increase the incidence of benign and malignant thyroid nodules (45). Breasts are known as the most radiosensitive organ in the human body (46). In the cervical radiology examination, breasts are exposed to scattered radiation, and the application of shielding reduces the dose by 88% in the current study. Studies highlighted the importance of minimizing radiation exposure to breasts in general radiography (47). Therefore, shielding can be applied if breasts are exposed to scatter radiation and positioned outside the primary field. On the other hand, shielding demonstrated dose reduction by almost 50% and more than 32% for heart and brain tissue, respectively.

Brain tissue is usually not protected due to the misrepresentation of its radio-resistance. Exposure to low doses of ionizing radiation may lead to stochastic effects, including neuro-vascular and neuro-degenerative effects and eye cataracts (48). The United Nations Scientific Committee on the Effects of Atomic Radiation (UNSCEAR) (49) and the International Commission on Radiological Protection (ICRP) (50) recommend particular attention to non-cancer diseases such as cataracts and changes in the cardiovascular system. The current results showed that the radiation dose to the skeletal tissue was reduced in a range from 37.2 to 10.6%. Exposure to ionizing radiation is associated with leukemia and bone cancer (51). Increased fracture risk was observed in bone after exposure to ionizing radiation (52). Nevertheless, the pancreas, spleen, kidneys, adrenal glands, and liver were away from the field of radiation, but the application of shielding reduced their doses by 7.9, 7.6, 4.8, 3.7, and 3.6%, respectively. A previous study showed that the application of lead-rubber inferolateral to the light beam diaphragm during arm radiography reduced radiation dose to the thyroid, left ovary, testes, left breast, and spleen by 13, 9, 6, 3, and 2%, respectively (47). The use of shielding over the organ showed a higher dose reduction in studies using anthropomorphic phantom and radiation dosimeters (9, 53). Nevertheless, the application of shielding in the current study was directly over the organ, leading to a higher radiation dose reduction than previous studies. Moreover, detailed analysis of dose reduction to many organs and tissues was an advantage.

## Conclusion

The frequency of radiological diagnostic procedures increased in recent years and will continue to increase every year (53, 54). The cumulative risk from multiple radiological examinations is a major concern, particularly for children and young patients (16). Efforts should be made to ensure patient radiation dose is always kept as low as reasonably achievable. The recommendation of the ICRP, dose optimization should be utilized when appropriate. Therefore, the use of shielding should be based on studies examining the effectiveness of shielding in reducing patient dose for particular radiology examinations as one size does not fit all. The MCNPX-MIRD optimized phantom provides detailed dose analysis for target and non-target organs and can be used to assess the efficiency of shielding in radiological examination.

## Data Availability Statement

The original contributions presented in the study are included in the article/supplementary material, further inquiries can be directed to the corresponding author/s.

## Ethics Statement

Ethics Institutional Review Board approval was not required because our work is based on phantom experimentation. Written informed consent was not required for this study because our work is experiment. No human experimentation was done.

## Author Contributions

WE, HT, HZ, and SI were responsible for conceptualization. MA and HT developed the methodology. HT, HZ, MA, and AE contributed software. SI, WE, and AE undertook validation. HZ, WE, and BI performed formal analysis. WE and HT assisted with the investigation. MA and BI resources. HZ, SI, and AE data curation. MA, WE, HT, and BI writing—original draft preparation. HZ, SI, and AE undertook writing—review and editing. WE and MA performed visualization. HT and BI were responsible for supervision. WE, HT, and SI contributed to project administration. AE undertook funding acquisition. All authors have read and agreed to the published version of the manuscript.

## Funding

The APC was supported by Dunarea de Jos University of Galati, Romania.

## Conflict of Interest

The authors declare that the research was conducted in the absence of any commercial or financial relationships that could be construed as a potential conflict of interest.

## Publisher's Note

All claims expressed in this article are solely those of the authors and do not necessarily represent those of their affiliated organizations, or those of the publisher, the editors and the reviewers. Any product that may be evaluated in this article, or claim that may be made by its manufacturer, is not guaranteed or endorsed by the publisher.
